# Live well: a practical and effective low-intensity dietary counseling intervention for use in primary care patients with dyslipidemia - a randomized controlled pilot trial

**DOI:** 10.1186/1471-2296-14-59

**Published:** 2013-05-12

**Authors:** Doina Kulick, Robert D Langer, Judith M Ashley, Kim M Gans, Karen Schlauch, Chad Feller

**Affiliations:** 1 Department of Internal Medicine-Reno, University of Nevada School of Medicine, 1500 E. 2nd Street, Suite #302, Reno, NV 89502, USA; 2 Department of Family Medicine-Las Vegas, University of Nevada School of Medicine, Las Vegas, NV 89012, USA; 3 Jackson Hole Center for Preventive Medicine, Jackson, WY 83002, USA; 4 Department of Agriculture, Nutrition and Veterinary Science, Dietetic Program, University of Nevada-Reno, Reno, NV 89557, USA; 5 Department of Behavioral and Social Sciences, Brown University, Providence, RI 02912, USA; 6 Brown University Institute for Community Health Promotion, Providence, RI 02912, USA; 7 Department of Biochemistry and Molecular Biology, University of Nevada-Reno, Reno, NV 89557, USA; 8 Nevada Bioinformatics Institute, University of Nevada-Reno, Reno, NV 89557, USA

**Keywords:** Diet, Behavior change, Lipid management, Cardiovascular, Risk reduction, Primary care

## Abstract

**Background:**

Diet is the first line of treatment for elevated cholesterol. High-intensity dietary counseling (≥360 minutes/year of contact with providers) improves blood lipids, but is expensive and unsustainable in the current healthcare settings. Low-intensity counseling trials (≤ 30 minutes/year) have demonstrated modest diet changes, but no improvement in lipids. This pilot study evaluated the feasibility and the effects on lipids and diet of a low-intensity dietary counseling intervention provided by the primary care physician (PCP), in patients at risk for cardiovascular diseases.

**Methods:**

Six month study with a three month randomized-controlled phase (group A received the intervention, group B served as controls) followed by three months of intervention in both groups.

Sixty-one adults age 21 to 75 years, with LDL-cholesterol ≥ 3.37 mmol/L, possessing Internet access and active email accounts were enrolled. Diet was evaluated using the Rate-Your-Plate questionnaire. Dietary counseling was provided by the PCP during routine office visits, three months apart, using printed educational materials and a minimally interactive counseling website. Weekly emails were sent reminding participants to use the dietary counseling resources. The outcomes were changes in LDL-cholesterol, other lipid subclasses, and diet quality.

**Results:**

At month 3, group A (counseling started at month 1) decreased their LDL-cholesterol by −0.23 mmol/L, (−0.04 to −0.42 mmol/L, P = 0.007) and total cholesterol by −0.26 mmol/L, (−0.05 to −0.47 mmol/L, P = 0.001). At month 6, total and LDL-cholesterol in group A remained better than in group B (counseling started at month 3). Diet score in group A improved by 50.3 points (38.4 to 62.2, P < 0.001) at month 3; and increased further by 11.8 (3.5 to 20.0, P = 0.007) at month 6. Group B made the largest improvement in diet at month 6, 55 points (40.0 to 70.1, P < 0.001), after having a small but significant improvement at month 3, 22.3 points (12.9 to 31.7, P < 0.001). No significant changes occurred in HDL-cholesterol in either group.

**Conclusions:**

A low-intensity dietary counseling provided by the PCP in patients at risk for cardiovascular diseases produced clinically meaningful improvements in both diet and lipids of magnitude similar to changes reported with high intensity interventions.

**Trial registration:**

ClinicalTrials.gov: NCT01695837

## Background

Approximately 16% of U.S. adults have unhealthy total cholesterol levels of 5.18 mmol/L or higher [1]. A sustained reduction of 0.6 mmol/L in total serum cholesterol level—an average decrease of 10%—can reduce coronary heart disease by about 25-30% [2]. Diet is the first line of treatment for high cholesterol [3]. For adult patients with hyperlipidemia and other known risk factors for cardiovascular and diet-related chronic disease, the current U.S. Preventive Services Task Force (USPSTF) clinical guidelines recommend high-intensity (more than 360 minutes/year of contact with providers) dietary counseling. Intensive counseling could be delivered by primary care clinicians or by referral to other specialists, such as nutritionists or dietitians [4]. Nonetheless, high-intensity and even medium-intensity (>30 and <360 minutes per year) counseling require resources that may not be available in most primary care settings in the U.S. Because of this reality, the current guidelines on dietary counseling for patients with high cholesterol are an elusive goal in contemporary clinical practice. Data from the 2010 National Ambulatory Medical Care Survey show that only 11.7% of visits to primary care physicians (PCP) included any type of counseling (provided by the PCP or referred out) for diet or nutrition. This percentage is down from 13.5% in 2006 [5,6].

Trials of low-intensity dietary counseling (≤ 30 minutes/year of provider contact) have found modest changes in diet, but failed to show improvement in lipid profiles [7]. Accordingly, this strategy is not currently recommended for use in clinical practice. While the high and medium intensity counseling intervention trials were conducted in subjects with hypercholesterolemia and other risk for cardiovascular diseases, most studies of low-intensity dietary counseling interventions were conducted in the general-risk population. The unselected population, along with other factors related to the design and delivery of the low-intensity interventions, may have contributed to the lack of significant impact of these interventions on cardiovascular risk reduction.

Innovative, time-efficient, and effective low-intensity strategies for dietary changes are needed, and clinical trials of these strategies are essential to justify their utility [7]. We hypothesized that a low-intensity dietary counseling intervention targeting patients with hypercholesterolemia and other cardiovascular risks deliverable within the context of a clinical visit would be a practical and effective option for use in primary care settings.

To test the feasibility and the effects of this strategy we designed a low-intensity dietary counseling intervention (LiveWell) to be delivered in the course of a primary care visits. The LiveWell intervention was conducted as a pilot study that enrolled patients with dyslipidemia who also scored low on a brief food- frequency screening questionnaire (Rate-Your -Plate) [8]. The counseling requires only 5–6 minutes of contact with the provider (in this case the primary care physician), and can be delivered during a regular office visit for dyslipidemia. This allows the physician to emphasize that diet is an integral part of dyslipidemia treatment and cardiovascular risk reduction. The intervention uses patient educational materials (paper and web-based) and weekly no-reply email reminders that prompt patients to pursue their dietary goals. This article reports the changes in LDL-cholesterol, other lipid subclasses, and diet quality produced by the LiveWell low-intensity dietary counseling intervention.

## Methods

### Study design

This was a six month pilot study with a three month randomized-controlled phase (during which group A received the intervention, and group B served as controls) followed by three months of intervention in both groups. The study was conducted at the University of Nevada, School of Medicine at Reno, from July 2011 through May 2012. The study used a 1:1 ratio simple randomization. The subjects’ allocation to groups was concealed by enclosing the assignments in serially numbered, opaque, sealed envelopes. The randomization list was computer generated. The envelopes were prepared by a statistician who was not involved with enrollment or data analysis.

### Participants

The population consisted of adults between the ages of 21 to 75 years, who received their health care at one of the University-affiliated primary care clinics. Eligibility required documentation of an LDL-cholesterol level of 3.37 mmol/L or higher within the twelve months prior to enrollment. Potential subjects were also required to have access to the Internet and an active email account. The use of lipid lowering drugs was not an exclusion criterion, but patients taking these drugs had to be on the same medication and dose for at least three months prior to enrollment, with no changes anticipated for the duration of the trial. We excluded patients with poorly controlled diabetes mellitus (defined by HbA1c > 9%), serum creatinine above 132.6 μmol/L, malignancy, cirrhosis, eating disorders, acute coronary syndrome in the last three months, congestive heart failure NYHA class III and IV, ongoing warfarin therapy, uncontrolled hypo- or hyperthyroidism, ongoing weight loss, history of bariatric surgery, pregnant women, and patients who scored more than 250 (on a scale of 100 to 300) on a food frequency questionnaire administered on the day of enrollment. Patients with a history of triglycerides >4.52 mmol/L were also excluded, because we used the Friedewald formula [LDL-C = Total cholesterol-(TGs/5 + HDL-C)] to calculate LDL-cholesterol levels. The study was conducted under the guidelines of the Declaration of Helsinki. The University of Nevada Institutional Review Board reviewed and approved the protocol. All subjects provided written informed consent before participation.

### Study protocol

**Figure 1:**
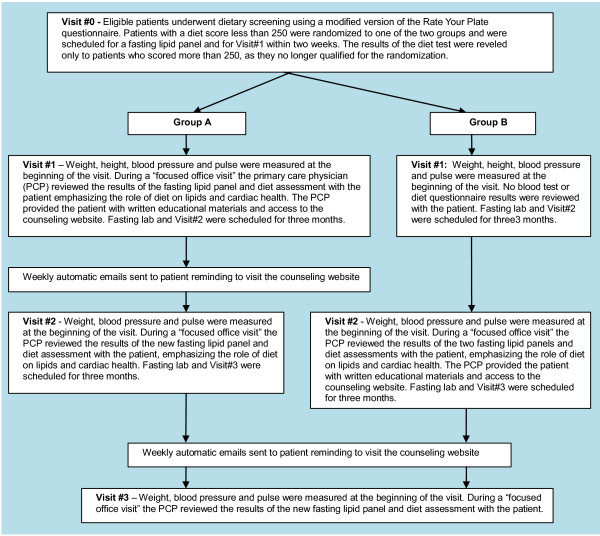
The outline of the study procedures.

Patients who met the LDL-cholesterol inclusion criteria were sent a letter to inform them about the study. Patients who responded to the letter were further assessed for the eligibility. The outline of the study procedures is shown in Figure 1.

#### The dietary assessment

Diet was assessed using a version of the Rate-Your-Plate (RYP) questionnaire modified for web-based use. The RYP was developed by Gans et al. in the late 1980s (periodically updated thereafter) as a paper-based, self-administered assessment tool of the eating-pattern that would allow a quick evaluation of dietary habits related to heart disease prevention [8,9]. This tool has been validated and shown to be an effective part of a program to lower patient’s cholesterol [10]. We used a modified version of the 2009 edition which has 25 questions. The answers in this questionnaire are displayed in three columns: column A includes the least “heart-healthy” choices; column C includes the most “heart-healthy” choices; and Column B is a “middle ground”. The RYP assigns points to these answers (1 point for answers in column A, 2 points and 3 points for the answers in column B and C, respectively. The sum of all the points produces an overall total diet score (on a scale of 25 to 75). This overall diet score is separated into tertiles that define three levels of overall diet quality: A = least heart healthy, C = heart healthy and B = in -between diet quality.

**Figure 2:**
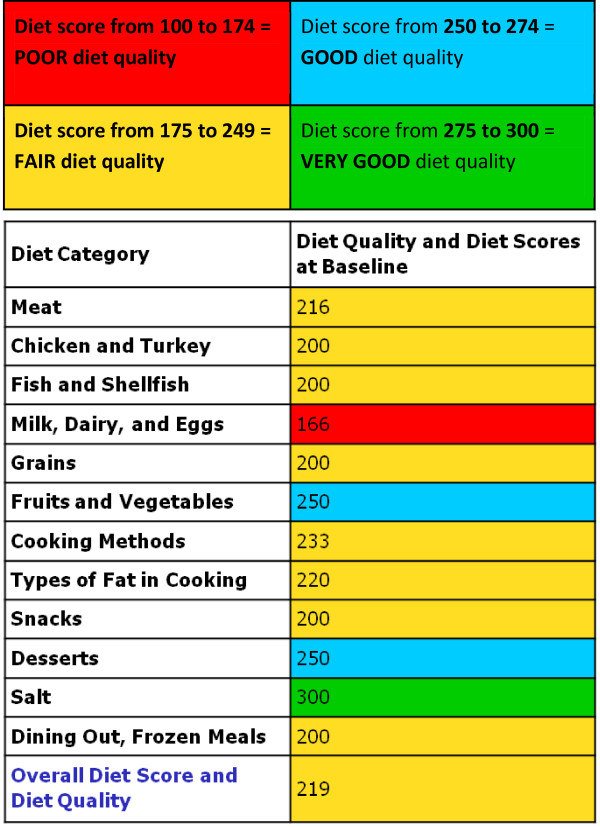
The computer generated display of the “Rate your Plate” Questionnaire Results.

While the RYP was the most suitable questionnaire that we could find to assess diet behavior in the primary care setting, the time needed to review each of the 25 items with the patient was too long to fit within a routine 15 minute physician office visit. In addition, the display of results in the original does not allow for easy spotting of “problem” items. To overcome these limitations we modified the display of the results. First, we consolidated the results of the 25 items into 12 “diet categories” by combining the items that would be naturally addressed together (e.g., items reflecting meat quality and meat portion size) and averaging the points of the corresponding items for each diet category. The RYP 25 to 75 scale was changed to a 100 to 300 scale that avoided decimal scores upon averaging. As mentioned, the original RYP categorizes the total diet score into three levels: A, B, and C. Using this scale, a patient’s overall diet could be classified as C = heart healthy while having a significant number of items answered as B. As our goal was to motivate patients to reach the best diet to lower cholesterol, we differentiated between the lower and the upper halves of the C = heart healthy overall diet scores and obtained four diet quality levels (A, B, C1 and C2). Taking advantage of computer graphics, we also created a color coded display of the results with red (poor diet) for A, yellow (fair diet) for B, blue (good diet) for C1 and green (very good diet) for C2 diet choices. The color coded display and the shorter list (12 diet categories instead of 25 items) provided an easy to interpret “snap-shot” of the patient’s diet (Figure 2). These innovations were possible by translating the paper version of the RYP into a self-administered computer-based version, which the patients could complete while in the physician’s office waiting room or at home. For this pilot study we administered the questionnaire at the study site to observe whether patients had questions while taking the test, and to monitor for technical difficulties.

#### The counseling materials

**Figure 3:**
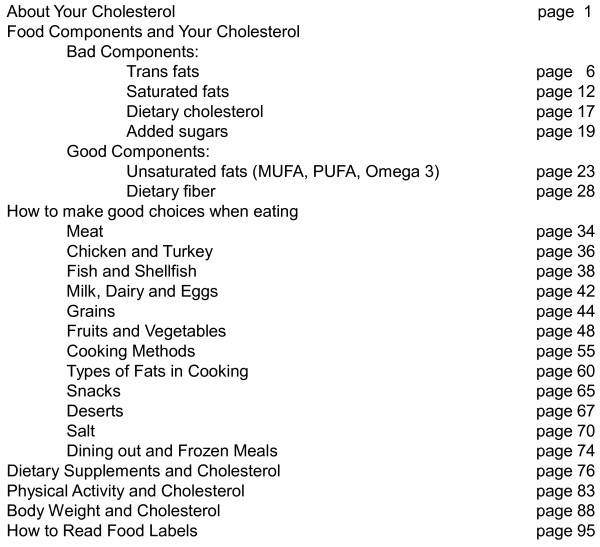
The table of contents for patient’s book on healthy diet and cholesterol.

We used paper-based patient educational materials and a secure counseling website. The paper-based patient educational materials consisted of a book specially written for this intervention. The book has 98 pages, with an average of 320 words per page, typed in size 11 font, with line spacing of 1.15. The average Flesch–Kincaid reading ease score of the book is 60, which should be easily understandable by 13- to 15-year-old students [11]. Figure 3 displays the Table of Contents. In addition to general information about the effect of diet, body weight and physical activity on cholesterol, the book has 12 sections corresponding to the 12 diet categories of the questionnaire report. The counseling website contains the same educational materials but adds an interactive, personal, password-protected section that includes the results of the patient’s diet test, their personal diet goals and provides tracking of their dietary changes. Access to the website was controlled by the website administrator (in this case the study investigator) in order to preserve the design of the study. The interactive section of the website allowed patients to set goals to improve their diet category scores. Once a patient selected one or more diet categories they wanted to improve, the interactive system would display a list of specific steps to achieve those goals (i.e.: 1- Trim all visible fat on the meat you eat; 2 - Use only 95% or leaner ground meet; 3 – Limit the portion size to 3–4 oz of meat per meal; etc.). Points were assigned to each step, and the total cumulative goal was computed and displayed. Completion of each step earned the patient one point. If the patient had not yet taken a particular step, but was planning to start doing it in the near future, a value of 0.5 points was assigned. Steps that the patient did not intend to take, or that s/he started but then stopped, were assigned a zero value. Weekly automatic, no-reply email reminders were sent to patients encouraging them to access the website, take the steps required and maximize their points to improve their diet. No additional patient counseling support (in person, by phone or email) was provided.

### Data collection

At baseline, data were collected on demographic characteristics (age, sex, years of education, use of Internet), height, weight, blood pressure, smoking, alcohol intake, cardio-metabolic past medical history, use of medications, diet assessment scores, and laboratory values. Weight was measured using a calibrated electronic scale and was recorded in pounds (to the nearest half pound). Height was measured using a stadiometer affixed to the wall, and was recorded in inches (to the nearest quarter inch). These measurements were obtained with subjects barefoot and wearing light garments. Body mass index (BMI) was calculated from these measurements as weight in kg/height in m^2^. Blood pressure was measured using a calibrated automatic sphygmomanometer, after the subjects were seated for five minutes. Blood samples for chemistries were drawn after a 12 hour overnight fast. The lipid panel was tested by LipoScience Inc., (Raleigh, NC) using nuclear magnetic resonance spectroscopy (NMR) to assess the lipid subclass particle counts and sizes, and standard chemical methods (Beckman Coulter and performed on Olympus AU Systems) to measure total cholesterol, HDL-cholesterol and triglycerides. LDL-cholesterol was calculated using the Friedewald formula [LDL-C = Total cholesterol-(TGs/5 + HDL-C)]. Fasting blood glucose was measured using a commercial enzymatic assay at the local LabCorp facility. Framingham risk score was calculated by using the regression equation from D’Agostino 2008 [12].

### Statistical methods

The sample size was chosen on the theoretical basis that at least 30 subjects per treatment group is reasonable for use of a parametric two-sample t-test to estimate treatment differences, assuming a normal distribution of the outcome measures [13].

Statistical analyses were conducted using SPSS for Windows version 12.0 (SPSS Inc. SPSS for Windows, Release 12.0.1. Chicago, IL, 2003). All p-values were two-tailed with p < 0.05 required for statistical significance. Frequencies were obtained for categorical variables. Differences between groups were tested using Chi-squared statistics in the Crosstabs procedure. For continuous variables, the UniANOVA procedure was used to compute means at baseline by group and to test between group differences at baseline without consideration of covariates. Correlations between the two overall diet scores (original and modified) were computed using the Partial Corr procedure with adjustment for age, sex, education in years and baseline BMI. Differences between the groups in mean changes between visits were assessed using the ANOVA procedure. The GLM procedure was used to perform repeated measures ANOVA with analyses stratified by treatment group to assess the changes in outcome variables between study visits 1 and 2, visits 2 and 3, and visits 1 and 3. This procedure reflected the study design under which Group A was expected to make significant changes between visit 1 and visit 2, then preserve or increase those changes at visit 3, while Group B was expected to make modest changes between visit 1 and visit 2, but greater changes between visit 2 and visit 3. These models were adjusted for age, sex, education in years and baseline BMI. For these models we report mean changes between visits, with 95% confidence intervals. The analysis was done by original assigned groups. The primary end points were the changes in LDL-cholesterol across time in the two groups. The secondary outcomes were changes in total cholesterol, HDL-cholesterol, triglycerides and diet score across time.

## Results

### Patients

**Figure 4:**
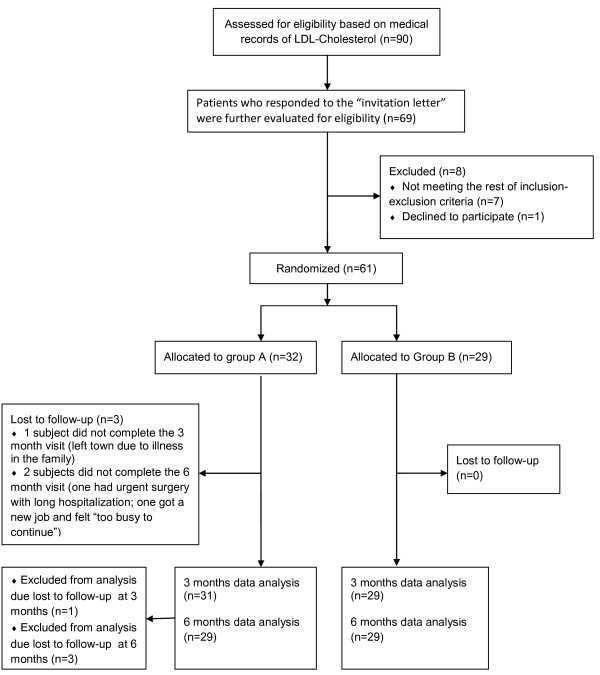
Study flow diagram.

Sixty-one subjects were enrolled and randomized to group A (N = 32) and B (N = 29); 95% completed the study (Figure 4). Three participants were lost to follow-up: one participant (group A) dropped from the study at visit 2 (had blood labs done but did not complete the clinical encounter) due to a family emergency; and two participants (group A) did not complete any elements of visit 3 (one was hospitalized for non-elective surgery and was unable to return in time to complete the study, and the other dropped out because he “got too busy”). Baseline characteristics by study group are presented in Table 1. Except for race, there were no significant differences between groups in baseline characteristics. The mean age was 52 years old, and the majority of the participants were white and female. The average BMI was 31. Most patients used the Internet at least 4 days a week. The average diet score (on the original RYP scale of 25 to 75, with 25 being the worst) was 50 (95% CI, 42.5 to 57.5) in group A, and 48 (40 to 56) in group B, P = 0.42; the corresponding modified diet scores were 201 and 193. The average baseline LDL-cholesterol in was 3.31 mmol/L (2.48 to 4.14 mmol/L) in Group A, and 3.37 mmol/L (2.87 to 3.86 mmol/L) in Group B, P = 0.71.

**Table 1:** **Characteristics of the patients at baseline**^a^

**Variable**	**Group A (N = 32)**	**Group B (N = 29)**	**P value**
Age (yr)	52.3 (±12.8)	52.1 (±12.7)	0.81
Sex (Female)	25 (78.1%)	21 (72.4)	0.80
Race			
White	22 (68.8%)	27 (93.1)	
Hispanic	7 (21.9%)	2 (6.9%)	0.048*
Asian	3 (9.3%)	0	
Years of education (yr)	14.7 (±2.6)	15.4 (±2.4)	0.39
BMI (kg/m^2^)	30.6 (±5.6)	31.4 (±7.1)	0.70
Internet use			
4-7 days/week	25 (78.1%)	26 (89.7%)	0.25
1-3 days/week	4 (12.5%)	0	
< 1 day/week	3 (9.4%)	3 (10.3%)	
Past medical hystory^b^			
A	6 (18.8%)	9 (31%)	
A,B	1 (3.1%)	0	
A,C	1 (3.1%)	0	0.56
A,D	1 (3.1%)	0	
B	2 (6.2%)	1 (3.4%)	
E	21 (65.6%)	19 (65.5%)	
Statins use (yes)	4 (12.5%)	1 (3.4%)	0.20
Lipid lowering dietary			
supplements	13 (40.6%)	6 (21%)	0.093
Yes			
Alcohol intake (drinks/day)			
None	21 (65.6%)	18 (62.1%)	
Less than one	4 (12.5%)	5 (17.2%)	
One	7 (21.9%)	4 (13.7%)	0.78
Two	0	2 (6.9%)	
More than two	0	0	
Smoking (yes)	2 (6%)	3 (10%)	0.56
Systolic blood pressure (mmHg)	123 (±12)	124 (±14)	0.92
Diastolic blood pressure (mmHg)	78.2 (±12)	78.4 (±15)	0.55
Diet quality (new format)			
Very good	0	0	
Good	0	1 (3.4%)	0.10
Fair	29 (90.6%)	20 (69.0%)	
Poor	3 (9.4%)	8 (27.6%)	
Diet quality (old format)			
A-Good	2 (6.3%)	3 (10.3%)	0.12
B-Fair	27 (84.4%)	19 (65.5%)	
C-Poor	3 (9.4)	7 (24.1%)	
Diet score (100 to 300 scale)	201 (±32)	193 (±35)	0.36
Diet score (25 to 75 scale)	50 (±7.5)	48 (±8)	0.42
LDL-particles number	2060 (±559)	2032 (±505)	0.82
Total cholesterol (mmol/L)	5.62 (±0.74)	5.78 (±0.56)	0.42
LDL-cholesterol (mmol/L)	3.31 (±0.84)	3.37 (±0.49)	0.71
HDL-cholesterol (mmol/L)	1.38 (±0.30)	1.46 (±0.32)	0.30
Total/HDL-cholesterol ratio	4.07 (±0.99)	4.17 (±0.99)	0.85
Triglycerides (mmol/L))	2.05 (±1.55)	2.06 (±0.99)	0.92
Fasting Glucose (mmol/L)	5.29 (±0.84)	5.16 (±0.56)	0.52

^a^ Continuous variables are given as mean and ± SD; categorical and ordinal variables data are given as number and (%).

^b^ A = Hypertension, B = diabetes mellitus type 2, C = coronary heart disease, D = cerebo-vascular accident, E = no past medical history of cardio-metabolic diseases.

### Diet

There was a very strong correlation between the “original” and “modified” diet scores, r = 0.967, p = 0.0005. Preliminary analyses showed the relationship to be linear, with both variables normally distributed, as assessed by the Shapiro-Wilk test (*P* >0.05), and there were no outliers.

Both groups improved their diet across time (Table 2). As expected, participants in group A (counseling started at visit 1), made the largest improvements in their overall diet scores between visit 1 and visit 2 (3 months later), then retained or continued to improve their diet at visit 3 (6 months from visit 1). The average diet score in group A at visit 1 was 203, (191 to 215) with an improvement of 50.3 (38.4 to 62.2, P < 0.001) at visit 2; and a further increase of 11.8 (3.5 to 20.0, P = 0.007) at visit 3. Also as predicted, Group B (counseling started at visit 2) made the largest improvement in their diet between visit 2 and visit 3, 55.0 (40.0 to 70.1, P < 0.001). Group B also had a small, but statistically significant improvement in overall diet score between visits 1 and 2; 22.3 (12.9 to 31.7, P < 0.001).

**Table 2:** The diet, lipid profile, BMI, and framingham risk score across time in the two groups (Models adjusted for age, sex, education, and baseline BMI)

**Outcome variable**	**Time**	**Group A**		**Group B**	
		**Adjusted means (95% CI) and changes in adjusted means (95% CI)**	**P values**	**Adjusted means (95% CI) and changes in adjusted means (95% CI)**	**P values**
Overall Diet Score (on the original scale of 25 to75)	V1	50.9 ( 48.1 to 53.8)		48.4 (45. 3 to 51.4)	
	V2	62.9 (60.6 to 65.1)		53.0 (49.6 to 56.4)	
	V3	65.2 (60.6 to 66.9)		66.1 (64.0 to 68.2)	
	V2 vs.V1	11.99 (9.2 to 14.7)	P < 0.001	4.7 (2.4 to 6.9)	P < 0.001
	V3 vs.V2	2 (0.1 to 4.4)	P < 0.001	13.1 (9.5 to 16.7)	P < 0.001
	V3 vs.V1	14.2 (11.1 to 17.4)	P = 0.038	17.8 (14.4 to 21.1)	P < 0.001
Overall Diet Score (on the modified scale of 100 to 300)					
	V1	203 (191–215)		193 (180 to 206)	
	V2	253 (244–263)		215 (201 to 230)	
	V3	265 (258–272)		270 (262 to 278)	
	V2 vs.V1	50.3 (38.4 to 62.2)	P < 0.001	22.3 (12.9 to 31.7)	P < 0.001
	V3 vs.V2	11.8 (3.5 to 20.0)	P = 0.007	55.0 (40.0 to 70.1)	P < 0.001
	V3 vs.V1	62.1 (50.1 to 74.1	P < 0.001	77.3 (63.5 -91.2)	P < 0.001
BMI					
	V1	30.4 (28.4 to 32.4)		31.2 (28.4 to 34.1)	
	V2	29.9 (27.9 to 31.9)		31.1 928.4 to33.90	
	V3	29.8 (27.7 to 31.8)		30.3 (27.7 to 33.0)	
(kg/m^2^)					
	V2 vs.V1	−0.5 (−0.1 to −0.8)	P = 0.01	−0.1 (−0.4 to 0.2)	P = 0.55
	V3 vs.V2	−0.2 (−.1.1 to 0.1)	P = 0.12	−0.8 (−0.4 to −1.2)	P = 0.001
	V3 vs.V1	−0.6 (−0.2 to −1.1)	P = 0.006	−0.9 (−0.4 to −1.4)	P < 0.001
LDL-Particles	V1	2048.3 (1861.8 to 2234.8)		2032.3 (1838.4 to 226.3)	
	V2	1837.5 (1575.3 to 1999.7)		2114.6 (1941.9 to 2287.2)	
	V3	1772.7 (1597.5 to1947.8)		1912.7 (1752.5 to 2072.8)	
Number					
	V2 vs.V1	−210.8 (−371.50 to −50.0)	P = 0.012	82.2 (−65.0 to 229.5)	P = 0.26
	V3 vs.V2	−64.8 (−180.4 to 50.7)	P = 0.26	−201.9 (−315.4 to −88.5)	P = 0.001
	V3 vs.V1	−275.6 ( −411.8 to −139.4)	P < 0.001	−119.7 (−241.3 to 1.9)	P = 0.053
LDL-Cholesterol					
	V1	3.28 (2.96 to 3.60)		3.44 (3.26 to 3.61)	
	V2	3.14 (2.90 to 3.38)		3.56 (3.26 to 3.75)	
	V3	2.96 (2.67 to 3.25)		3.44 (3.14 to 3.73)	
(mmol/L)					
	V2 vs.V1	−0.14 (−0.43 to 0.15)	P = 0.38	0.12 (−0.04 to 0.29)	P = 0.14
	V3 vs.V2	−0.18 (−0.01 to −0.36)	P = 0.046	−0.12 (−0.38 to 0.14)	P = 0.40
	V3 vs.V1	−0.32 (−0.06 to −0.58)	P = 0.016	0.001 (−0.27 to 0.28)	P = 0.90
HDL-Cholesterol					
	V1	1.36 (1.26 to 1.45)		1.46 (1.36 to 1.56)	
	V2	1.42 (1.30 to 1.54)		1.46 (1.36 to 1.55)	
	V3	1.38 (1.27 to 1.49)		1.44 (1.34 to 1.53)	
(mmol/L)					
	V2 vs.V1	0.06 (−0.02 to 0.15)	P = 0.14	−0.01 (−0.06 to 0.05)	P = 0.82
	V3 vs.V2	−0.04 (−0.03 to 0.05)	P = 0.28	−0.02 (−0.08 to 0.05)	P = 0.54
	V3 vs.V2	0.02 (−0.03 to 0.08)	P = 0.37	−0.03 (−0.10 to 0.05)	P = 0.47
Triglycerides					
	V1	2.06 (1.58 to 2.54)		2.06 (1.68 to 2.44)	
	V2	1.68 (1.41 to 1.95)		1.95 (1.53 to 2.37)	
(mmol/L)	V3	1.85 (1.51 to 2.19)		1.65 (1.26 to 2.03)	
	V2 vs.V1	−0.38 (0.76 to 0.16)	P = 0.055	−0.11 (−0.27 to 0.05)	P = 0.16
	V3 vs.V2	0.17 (−0.16 to 0.50)	P = 0.30	−0.30 (−0.63 to 0.02)	P = 0.07
	V3 vs.V1	−0.21 (−0.65 to 0.24)	P = 0.34	−0.41 (−0.76 to −0.06)	P = 0.03
Total					
	V1	5.60 (5.34 to 5.85)		5.78 (5.58 to 5.97)	
	V2	5.35 (5.06 to 5.66)		5.87 (5.64 to 6.10)	
	V3	5.15 (4.83 to 5.46)		5.60 (5.27 to 5.92)	
Cholesterol					
(mmol/L)					
	V2 vs.V1	−0.24 (−0.49.1 to 0.01)	P = 0.06	0.10 (−0.09 to 0.28)	P = 0.30
	V3 vs.V2	−0.21 (−0.44 to 0.02)	P = 0.07	−0.27(−0.52 to −0.03)	P = 0.03
	V3 vs.V1	−0.45 (−0.65 to −0.25	P = 0.001	−0.18 (−0.49 to 0.15)	P = 0.25
Framingham risk score (%)					
	V1	9.2 (7.0 to 11.4)		8.4 (7.0 to 9.8)	
	V2	8.3 (6.3 to10.3)		7.8 (6.5 to 9.1)	
	V3	6.9 (5.1 to 8.7)		7.1 (6.0 to8.2)	
	V2 vs.V1	−0.9 (−1.5 to-0.3)	P = 0.005	−0.6 (−1.5to 0.3)	P = 0.19
	V3 vs.V2	−1.4 (−2.5 to −0.2)	P = 0.03	−0.7 (−1.2 to −0.2	P = 0.014
	V3 vs.V1	−2.3 (−3.6 to −0.9)	P = 0.002	--1.3 (−2.1 to −0.5)	P = 0.003

**Figure 5:**
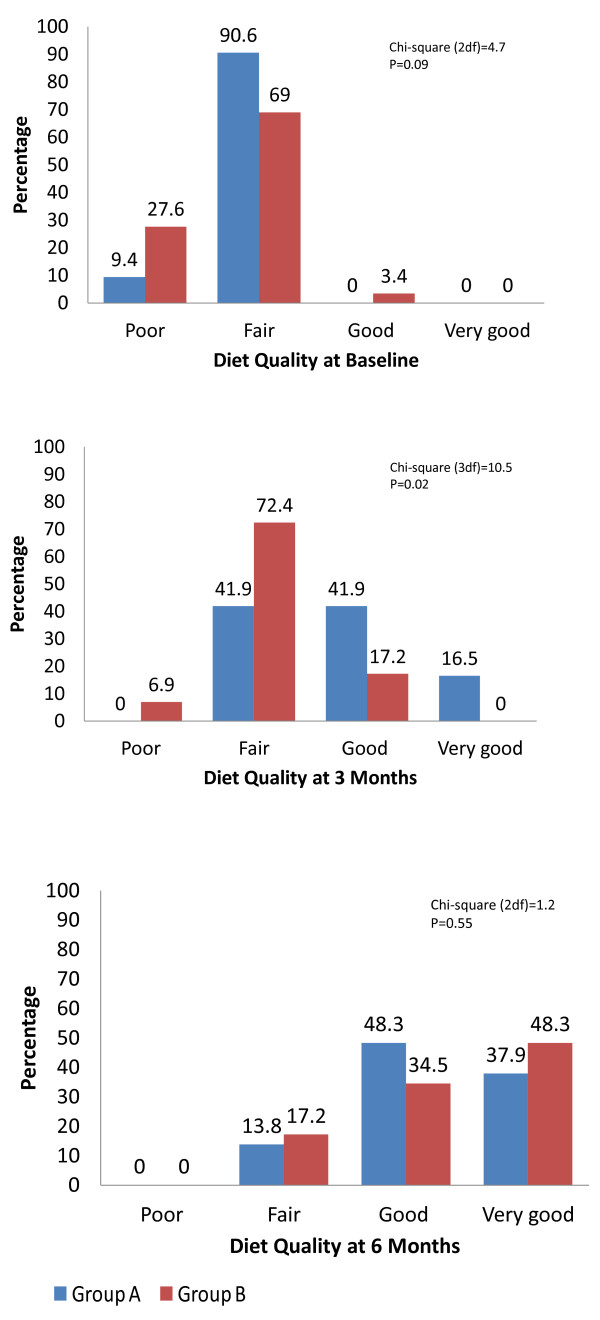
The percentages of diet quality categories (poor, fair, good and very good) in the two groups at: baseline (v1), three months (v2) and six months (v3).

Figure 5 displays the percentages for diet quality (poor, fair, good, and very good) in the two groups at baseline, three, and six months. At baseline there were no statistically significant differences between the groups, χ^2^(2) = 4.7, *P* = 0.09. At three months, diet quality in Group A improved significantly compared with group B, χ^2^(3) = 10.652, *P* = 0.013: there were no participants with poor diet in group A; 16.1% of them (vs. 3.4% in group B) achieved a very good diet; and 41.9% of group A had a good diet compared to 17.2% in group B. As predicted, at six months (3 months after dietary counseling was initiated in group B), there was no statistically significant difference between the groups, χ^2^(2) = 1.2, *P* = 0.5: there were no participants with poor diet scores in either of the two groups, and the percentage of participants that scored a very good diet was 37.9% in group A and 48.3% in group B.

### Lipids

As shown in Table 2, in group A changes from baseline in total and LDL-cholesterol were not significant at visit 2, but did become statistically significant at visit 3: total cholesterol −0.45 mmol/L (−0.65 to −0.25 mmol/L, P = 0.001) and LDL-cholesterol −0.32 mmol/L (−0.06 to −0.58 mmol/L, P = 0.016). In contrast, LDL-particle count decreased significantly at visit 2, -210.8 (−371.50 to −50.0, P = 0.012), and this improvement was maintained at visit 3. In Group B, which had the start of dietary counseling delayed to visit 2, the changes between visit 2 and visit 3 were statistically significant for LDL-particle number, -201.9 (−315.4 to −88.5, P =0.001) and for total cholesterol, -0.27 mmol/L (−0.52 to −0.03 mmol/L, P = 0.03), but the change in LDL-cholesterol was not statistically significant −0.12 mmol/L (95% CI, -0.38 mmol/L to 0.14 mmol/L), P = 0.4. Adjusted means for total-cholesterol, LDL-cholesterol and LDL-particle count at visit 2 were significantly better in Group A than Group B. Compared with group B, group A decreased their LDL-particle count by an average of −213.9, (−70 to – 357, P = 0.001; LDL-cholesterol by an average of −0.23 mmol/L, (−0.04 to −0.42 mmol/L, P = 0.007 and total cholesterol by −0.26 mmol/L, (−0.05 to −0.47 mmol/L, P = 0.001). At the six month visit, changes in group A remained better than those in group B for total and LDL-cholesterol, while there was no statistically significant difference between the groups in LDL-particle count.

In contrast to the significant changes in total and LDL-cholesterol, there were no significant changes over time in HDL-cholesterol in either group, and the triglycerides showed significant improvement only in group B at visit 3.

Body mass index declined at six months in Group A by an average of −0.6 kg/m2 (−0.2 to −1.1 kg/m2), P = 0.006, and in group B by −0.9 kg/m2 (−0.4 to −1.4 kg/m2), P < 0.001. The 10 years Framingham risk score showed also a small, but statistically significant improvement across time in the two groups, more prominent in group A than Group B. At six months patients in Group A lower there their risk score by an average of 2.3% (0.9% to 3.6%) and Group B by 1.3% (0.5% to 2.1%).

## Discussion

A meta-analysis performed in 2010 to assist the U.S. Preventive Services Task Force in formulating recommendations on physical activity and dietary counseling to prevent cardiovascular disease found that high-intensity counseling intervention on diet or combined lifestyle counseling decreased total cholesterol by 0.17 mmol/L (0.09 to 0.25 mmol/L) and LDL-cholesterol by 0.13 mmol/L, (2.39 to 0.21 mmol/L) [7]. The same meta-analysis found no statistically significant changes in HDL- cholesterol and triglycerides. High-intensity dietary counseling, with or without physical activity counseling, also resulted in changes of −0.3 to −0.7 kg/m^2^ in BMI. The low-intensity trials included in the above meta-analysis failed to show any benefits in the lipid profile, although there was some improvement in the healthful eating behavior.

Our low intensity dietary counseling intervention appears to be feasible in the settings of current primary care practice and produced outcomes comparable to those of high intensity intervention trials in both diet and lipids, and BMI. As hypothesized, statistically significant changes in diet were achieved in each group after the intervention. In group A, which started the intervention at visit 1, the most prominent improvement in diet was seen at visit 2, and continued to improve at visit 3. For Group B, which started the intervention at visit 2, the most prominent improvement occurred at visit 3. Interestingly, group B showed a smaller, but statistically significant improvement in diet at visit 2 as well. This could be the result of motivated patients who were interested in participating in lifestyles changes study, or could be due to a Hawthorn effect, both of which could underestimate the magnitude of the effect of the counseling intervention. Lipid levels (especially total and LDL–cholesterol) are known to have seasonal variations, with values being approximately 0.18 mmol/L higher in the winter than in the summer [14,15]. Participants in this study had baseline lipids drawn in July-September; the 3 month follow-up labs were drawn during November- January, and the six month follow up labs were drawn during February- May. Consistent with an effect of this nature, in group B there was a trend for higher total and LDL-cholesterol at visit 2 (occurring in winter and before they were exposed to the intervention) compared with baseline. The changes in these lipid fractions between the groups at visit 3 are the least biased by season since those assessments were done contemporaneously and this visit was conducted between February and May when dietary distortions due to the holidays were not a factor. Accordingly, these results represent the most accurate test of the effect of dietary counseling on total and LDL–cholesterol and showed significantly better outcomes in Group A.

While this was not a weight loss intervention, we did see a small, but statistically significant decrease in BMI across time in both groups. We also noticed a small and statistical significant improvement in the 10 years Framingham risk score. While these results are statistically significant they may not be clinically relevant in our opinion, due to their small magnitude.

Physicians are perceived by patients as the most credible source of health information [16]. Patients value the physicians’ advice, and are motivated to act on it [17]. Nonetheless, the reality is that during a routine office visit, the physician has only few minutes to assess a medical problem (reviewing a brief history and few diagnostic tests), and to initiate treatment (writing a drug prescription). This leaves very little time for dietary counseling [18,19]. In addition, some studies have found that physicians do not posses adequate nutritional knowledge to deliver efficient dietary counseling or they may lack confidence in doing so [20,21]. The counseling intervention we designed and pilot-tested in this study addresses these two major barriers. First, it requires very brief face-to face patient-provider time it so that it can be delivered during routine office visits for dyslipidemia. This allows physician to make diet an integral part of the management strategy for dyslipidemia and cardiovascular risk reduction. Second, the physicians’ nutrition counseling skills are enhanced by standardized written and web-based tools that allow the physician to provide patients with pertinent, clearly written dietary advice.

A major challenge faced in developing this intervention was the paucity of dietary screening questionnaires that could be used in primary care setting. We determined that the Rate-your-Plate questionnaire was the most appropriate tool for this purpose. We made several changes to the display of the diet scores, in order to facilitate a faster, easier review of the diet quality. It is important to note that our modified diet scores had nearly perfect correlation (Pearson correlation, r = 0.967, p = 0.0005) with the original scores.

This study has several limitations. First, we assessed diet changes using only self reported screening methods. Secondly, the study was only six months in duration. This relatively short interval of follow up, and the selection bias of highly motivated volunteers who were interested in making changes to their diet, could overestimate the effect of the intervention on the outcomes. Also, the physician delivering the intervention developed it, and was similarly highly motivated. Thus, generalization of this dietary counseling intervention requires further assessment in less ideal environments and in a wider range of patients and providers, with a longer time of follow-up. A larger sample size would allow for assessing the effect o the intervention in important subgroups of patients (based on socio-economic status, level of education, age groups, etc.). The current study uses both paper-based and internet-based counseling materials, which may be redundant. Additional study arms to explore the effect of the counseling using paper-based only and web-based only should be considered in a future larger sample size study.

## Conclusions

Efficient low-intensity dietary counseling provided by the PCP during routine office visits, as an integrative part of dyslipidemia treatment, is feasible by using written and web-based counseling tools. In this pilot study we observed clinically meaningful improvements in both diet and lipids of magnitude similar to changes reported with high intensity dietary counseling interventions. Further study in a larger more diverse population and with longer follow-up is warranted to validate these preliminary results. Finding efficient low-intensity dietary counseling interventions, sustainable in the current clinical settings, will help close the gap between clinical research and clinical practice.

## Abbreviations

PCP: Primary care physician; RYP: Rate your plate; BMI: Body mass index.

## Competing interests

All the authors declare that they do not have any competing interests.

## Authors’ contributions

DK designed the protocol, outlined and develop the content of the counseling tools, conducted the study, and coordinated the collection the clinical data. DK and RDL analyzed the data. JA and KMG contributed to the development of dietary assessment and of counseling materials. KS and CF helped develop the database and interactive web-based counseling system. All five authors have been involved in drafting the manuscript or revising it critically for important intellectual content and have given final approval of the version to be published. All authors read and approved the final manuscript.

## Pre-publication history

The pre-publication history for this paper can be accessed here:

http://www.biomedcentral.com/1471-2296/14/59/prepub
